# Urologist led one-stop testicular clinic: the UK 'gold standard’

**DOI:** 10.1186/s40064-016-1722-7

**Published:** 2016-01-29

**Authors:** David Muthuveloe, Nkwam Nkwam, Paul Hutton, D. M. A. Wallace, Richard Viney, Prashant Patel

**Affiliations:** School of Cancer Sciences, University of Birmingham, Edgbaston, Birmingham, UK; Nottingham City Hospital, Hucknall Rd, Nottingham, UK; Department of Urology, University Hospitals Birmingham, Birmingham, UK

**Keywords:** Testicular cancer, Rapid access testicular clinic, One-stop testicular clinic

## Abstract

Prompt diagnosis and early treatment for testicular cancer is vital. To help with this a one-stop, urologist run, testicular clinic with testicular ultrasound scanning as an integral part of the clinic format was introduced to investigate patients in an efficient and timely manner. The aim of this study was to assess the feasibility and efficiency of running a one-stop testicular clinic. A prospectively collected electronic database of all patients attending a one-stop testicular clinic at a busy university hospital was interrogated over a 6-year period. Only new referral males, above the age of 15 years old were included. Case notes were reviewed retrospectively. A total of 1757 patients were found with a median age of 36. 6.3 % had a suspicious ultrasound scan and overall 5.6 % were found to have malignancy histologically. In addition a significant proportion of men with a history of testicular maldescent went on to develop testicular cancer (p < 0.01). Median time from referral to clinic and clinic to orchidectomy for suspected testicular cancers was 9 and 5 days respectively (95 % CI). Some of the benefits of a urologist run one-stop testicular clinic include: timely diagnosis and treatment, early reassurance with normal investigations, the discovery of clinically unsuspecting malignancy and the increase in teaching opportunities. These collective benefits must improve patient experience and benefit the department as a whole. A urologist led one-stop testicular clinic should be regarded as the gold standard.

## Background

Testicular cancer accounts for approximately 1 % of all the malignancies found in the general male population (La Vecchia et al. 2010). In 2011 it was rated 16th most common male cancer in the UK (Office for National statistics 2013), however it is the most frequent cancer identified in men between the ages of 15–35. Over the last three to four decades the incidence of testicular cancer world wide has increased and in the UK the incidence has doubled (Huyghe et al. 2003; Albers et al. 2005). Reassuringly with effective treatment, testicular cancer is highly curable (Viatori 2012). Early diagnosis and treatment is a high priority in these cases and healthcare workers must be aware of the importance in education for the young male population. A one-stop testicular clinic was introduced to facilitate the investigation and treatment of patients in an efficient, timely and effective manner.

The aim of this investigation was to assess the feasibility and efficiency of running a one-stop testicular clinic.

## Urologist led one-stop testicular clinic

To help with rapid investigation and diagnosis, a urologist led one-stop testicular clinic with testicular ultrasound scanning was created. According to the United Kingdom national guidelines patients who are referred to a urologist with a possible malignant testicular mass must be seen within 2 weeks, however this does not necessarily mean that a diagnosis or treatment plan has been implemented. Often the first clinic appointment enables history and examination by a urologist but further investigations are often required. Blood tests and ultrasound scans are invariably needed to make a final diagnosis and treatment plan, which all require further clinic appointments.

The rationale of a urologist led one-stop testicular clinic was to expedite the patient’s journey through from referral to diagnosis and then to treatment (Kendall et al. 1996). Testicular ultrasound scanning forms an integral part of the clinic format. The key personnel required to run these clinics are a urologist who takes a detailed history and examination, and a testicular cancer specialist nurse. Facilities for phlebotomy and for testicular ultrasound scanning (to be done ideally by the urologist) have to be available and the appropriate patient literature on all testicular pathology needs to be present. This type of clinic format would result in a prompt patient journey from referral to diagnosis.

## Methods

A prospectively collected electronic database of all patients attending a one-stop testicular clinic at a busy university hospital was interrogated over a 6-year period. Only new referral males, above the age of 15 year old were included. Case notes were reviewed retrospectively. Patient risk factors, referral details and clinic letters were examined. Ultrasound results were compared to final histology results for concurrence. The testicular tumor markers alpha feto-protein (αFP), beta human chorionic gonadotropin (βhcg) and lactate dehydrogenase (LDH) were also analyzed.

## Results

A total of 1757 patients were seen over a period of 67 months. The overall detection rate for malignancy was 5.6 % (98 cases). The overall median age was 36 (15–92). In those with histologically proven malignancy the median age was 31 (17–82), which was significantly lower than those without malignancy (p < 0.05). 6 % of cases between the ages of 15 and 34 were found to have a malignancy compared to 3 % of cases that had a malignancy above the age of 34. Therefore there was almost twice the likelihood of being diagnosed with malignancy in the younger age group (RR 1.9). The median duration of symptoms was 8 weeks. There was almost equal split in laterality with 47 % of cases on the left, 45 % of cases on the right and 8 % of cases were bilateral.

Median waiting time to be seen for all patients was 13 days (95 % CI). For those with testicular cancer the median waiting time to be seen was 9 days (95 % CI). The median waiting time from clinic to orchidectomy was 5 days (95 % CI).

**Fig. 1:**
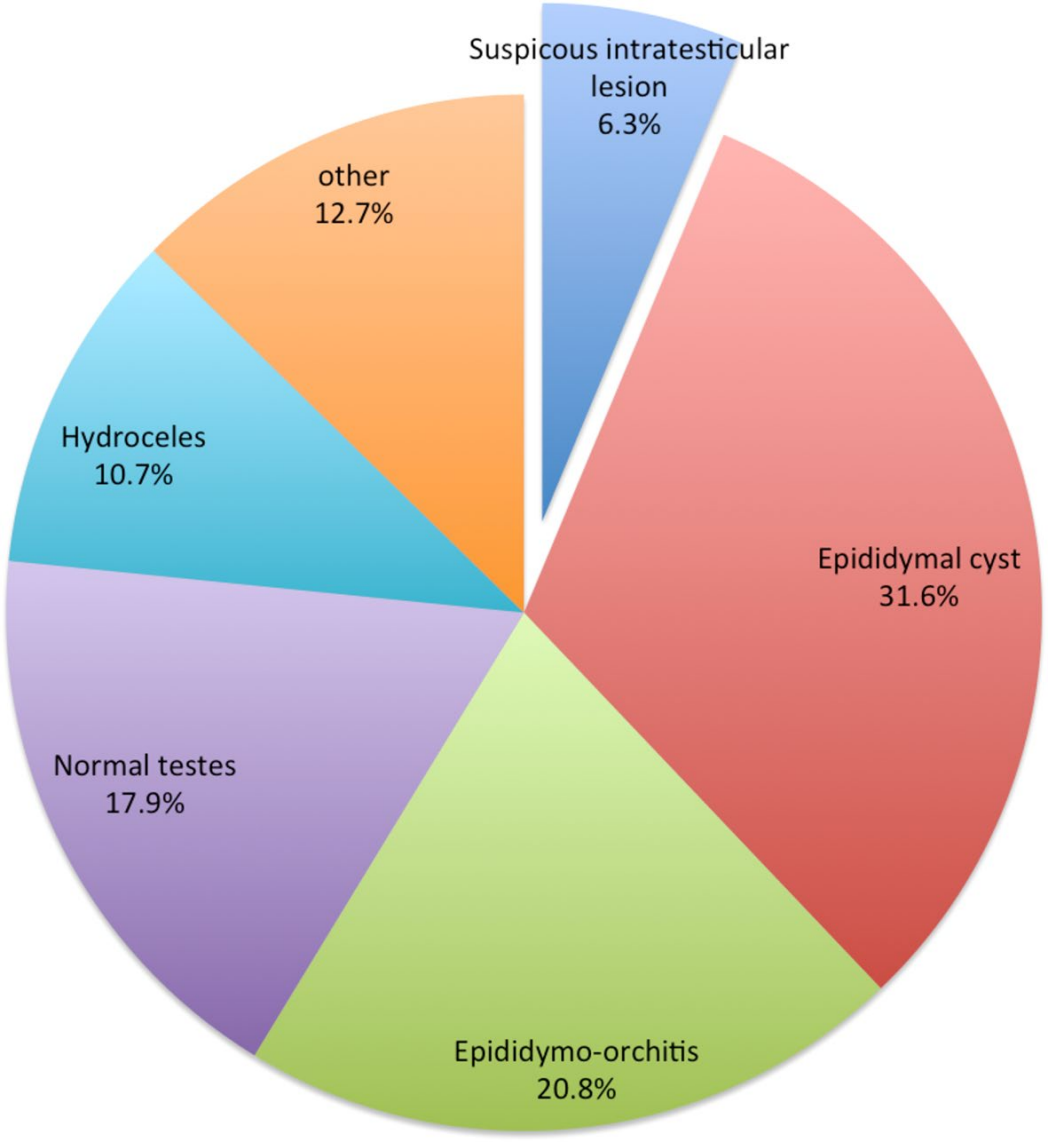
A chart describing all testicular ultrasound scans results

1740 (99 %) had testicular ultrasound scans. 109 cases (6.3 %) had an ultrasound scan that appeared suspicious of testicular cancer. 17.9 % had no abnormalities on ultrasound scan and the rest had benign lesions. The majority of these benign lesions were epididymal cysts (31.6 %), epididymo-orchitis (20.8 %) and hydroceles (10.7 %) (Fig. 1).

98 out of the 109 cases with abnormal ultrasound scans were subsequently found to have malignancy on histological examination. Therefore when compared to the ultrasound findings there were 11 false positives (Table 1).

**Table 1:** The histology results for those cases with a suspicious testicular ultrasound scan but no malignancy on histology

	Frequency
Epidermoid cyst	3
Tuberculous orchitis	2
Chronic active epididymoorchitis	2
Testicular microlithiasis	2
Not malignant	2
Total	11

**Fig. 2:**
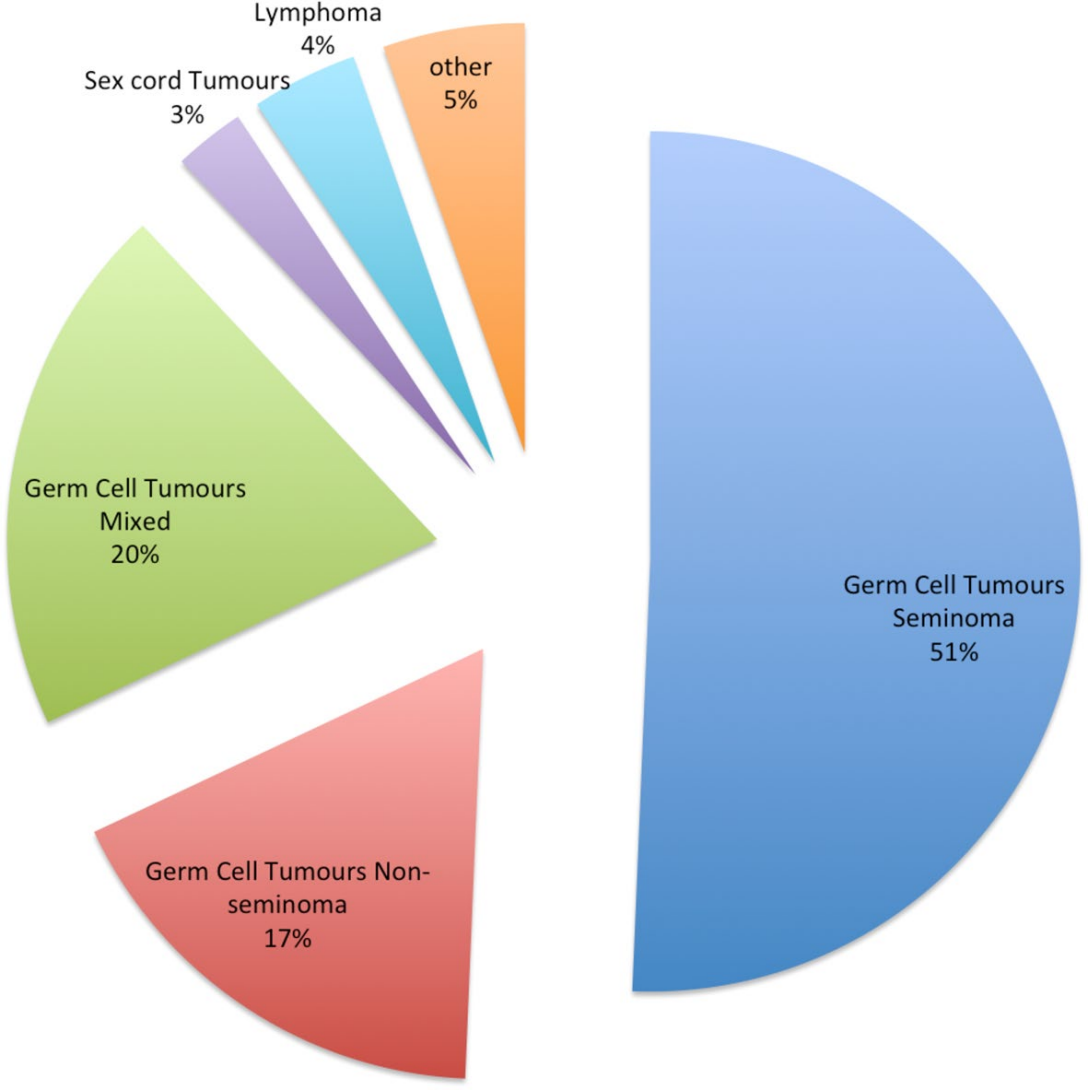
A pie chart showing all the types of malignancy found following orchidectomy

The most common histology result for those with malignancy was seminomatous germ cell tumor (51 %) as shown in Fig. 2.

5.7 % of the men with testicular cancer had a history of maldescended testes in the past. This compares with 1.4 % who did not have cancer who had a history of maldescended testes. As a result a significant proportion of men with a history of maldescended testicles went on to develop testicle cancer (p < 0.01) giving a relative risk of 4.1.

**Fig. 3:**
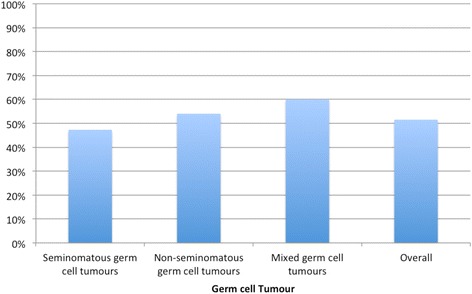
Proportion of germ cell tumor cases with positive testicular tumor markers (αFP, βhcg, LDH)

Overall 51.5 % of those with testicular germ cell tumors (GCT) were found to have a raised serum testicular tumor marker (αFP, βhcg or LDH). 47 % of seminomas, 54 % of NSGCT and 60 % of mixed GCT had at least one or more raised serum testicular tumor marker (Fig. 3).

## Discussion

Following the release by the UK National Health Service (NHS) Modernization Agency in 2004 (NHS Modernisation Agency 2004) of advice relating to service improvement, three of the ten high impact changes were particularly pertinent to these clinics. They included the help to improve access to key diagnostic tests, avoid unnecessary follow-ups and improve patient access by reducing the number of queues. Subsequently the rule that those with suspected cancer should be seen within 2 weeks became well established (Kendall et al. 1996). In urology the one-stop clinics was a natural follow-on and this has been established in those patients with suspected prostate cancer and those with hematuria. However the appetite for a one-stop testicular clinic has been lacking and has not been widely accepted.

There are many apparent benefits from having a rapid access one-stop testicular cancer clinic. Primarily it reduces the multiple attendances that a patient has to go through to get a diagnosis. This helps with faster diagnosis and treatment. In our group we found a median waiting time of those with testicular malignancy of 9 days and a median waiting time from clinic to orchidectomy of 5 days. Prompt diagnosis and scheduling for treatment must also help reduce the risk of complications and must benefit the department as a whole. In addition it helps alleviate patient anxiety in those with normal examinations and normal investigations.

The ease of access for general practitioners (GPs) to this service is useful and should be regarded as beneficial. However it could be argued that the improved service could increase referral rates, as some GPs may not be confident to reassure patients based on examination alone. This lack of confidence is not an unreasonable concern as approximately 2200 new cases of testicular malignancy are diagnosed each year in the UK (Cancer Research UK 2012), GPs may only see one or two cases of testicular malignancy in their lifetime. This is supported by a study from Gloucester that showed that up to 81 % of 2 week wait referrals to testicular clinic when subsequently examined by a specialist were thought to be inappropriate (Foster et al. 2006). As a result the improved service may stimulate demand. However our results show that the referral rates did not increase year on year but in fact remained stable. This is thought to be due to the reduction of clinic re-attenders caused by the prompt reassurance of a normal examination and ultrasound scans.

Routine testicular ultrasound is an integral part of the one-stop testicular clinic. A urologist who is trained in scrotal ultrasound scanning is needed for this service. This can be a controversial issue as not only is it taking work and possibly funding away from the radiology department; the radiology department may become deskilled in this area. This problem can be addressed by having an instant access ultrasonographer available alongside the clinic to do the scan; however in the clinic analyzed, an appropriately trained urologist performed the ultrasound scan.

Another controversial issue is the reliability of an ultrasound scan performed by a urologist. Ultrasound scrotum like any other ultrasound procedure is operator dependent and there may be some concern as to the accuracy of the result. However in this study we have shown that in the hands of an urologist trained in scrotal ultrasound there is excellent accuracy in diagnosing malignancy with a 90 % specificity rate. This is comparable to previously published literature (Polák and Hornák 1990; Guthrie and Fowler 1992). This means that with an appropriately trained urologist there is good evidence that the ultrasound results are robust and should be reliable.

The liberality of ultrasonography in these cases could be of some debate. In a one-stop clinic almost every patient had a testicular ultrasound scan (99 %) regardless of the examination findings. It could be argued that there was over-investigation in some cases especially if the clinical examination was normal or showed benign disease. In the normal situation if a urologist felt that the examination was normal or the patient had benign disease then a formal ultrasound scan may not necessarily have been requested. However we the authors disagree with this assumption. This is due to the fact that the examination findings are not always reliable and may miss small clinically unsuspecting disease. In addition there may be dual pathology. One study found that 1.3 % of patients with a clinical diagnosis of epididymal cyst (mainly by urologists) were found to have a malignancy on subsequent ultrasound scan (Guthrie and Fowler 1992). This shows that examination, although useful shouldn’t necessarily be relied upon completely and that as ultrasound scanning is quick, relatively cost effective and non-invasive it should be used.

A further advantage of this clinic set up is the excellent environment for urological trainees to gain concentrated experience in testicular ultrasound and for medical students to gain experience in male genital examination. Learning opportunities around these areas can be difficult to come across during training however this clinic would provide the ideal occasion to teach these key skills.

It is well established that cryptorchidism is a risk factor for testicular cancer (Kundra 2004). We found that a significant number of those with testicular cancer had a history of testicular maldescent (p < 0.01). A history of testicular maldescent was found to be a significant independent predictive marker of being diagnosed with testicular cancer with 4 out of the 22 (18 %) being diagnosed with testicular cancer when referred to testicular clinic. Several studies have shown the relative risk of testicular cancer in those with testicular maldescent ranges from 2.1 to 17.6 (Herrinton 2003) and our study supports this with a relative risk of 4.5 in those who were referred to testicular clinic.

## Conclusion

With a urologist led one-stop testicular clinic we have shown not only convenience for patients and referring doctors, but also a fast referral to clinic time and a fast referral to treatment time in those with suspected testicular cancer. This benefits patients and leads to less anxiety as diagnoses are made rapidly. In addition the reassurance of a normal or benign ultrasound scan would reduce the number of re-attenders. We have also shown that an appropriately trained urologist can perform testicular ultrasound scans with high accuracy and specificity comparable to previously published data. This saves time and also departmental funds. We believe that these collective enhancements are positive and benefit patients and the department as a whole. A urologist led one-stop testicular clinic should be regarded as the gold standard.
